# Deep learning-based rice pest detection research

**DOI:** 10.1371/journal.pone.0313387

**Published:** 2024-11-07

**Authors:** Peng Xiong, Cong Zhang, Linfeng He, Xiaoyun Zhan, Yuantao Han

**Affiliations:** Wuhan Polytechnic University, Wuhan, Hubei, China; Queensland University of Technology, AUSTRALIA

## Abstract

With the increasing pressure on global food security, the effective detection and management of rice pests have become crucial. Traditional pest detection methods are not only time-consuming and labor-intensive but also often fail to achieve real-time monitoring and rapid response. This study aims to address the issue of rice pest detection through deep learning techniques to enhance agricultural productivity and sustainability. The research utilizes the IP102 large-scale rice pest benchmark dataset, publicly released by CVPR in 2019, which includes 9,663 images of eight types of pests, with a training-to-testing ratio of 8:2. By optimizing the YOLOv8 model, incorporating the CBAM (Convolutional Block Attention Module) attention mechanism, and the BiFPN (Bidirectional Feature Pyramid Network) for feature fusion, the detection accuracy in complex agricultural environments was significantly improved. Experimental results show that the improved YOLOv8 model achieved mAP@0.5 and mAP@0.5:0.95 scores of 98.8% and 78.6%, respectively, representing increases of 2.8% and 2.35% over the original model. This study confirms the potential of deep learning technology in the field of pest detection, providing a new technological approach for future agricultural pest management.

## 1. Introduction

Agriculture not only supports human survival and development but also plays a fundamental role in societal progress [1]. However, crops are often severely threatened by pests, leading to yield reduction and quality degradation, which, in extreme cases, can even jeopardize food security [2, 3]. Although chemical pesticides have been widely used for pest control, they pose significant challenges to food safety and agricultural sustainability due to environmental pollution, negative impacts on beneficial insects, and residues in food products [4, 5]. Consequently, timely detection and effective control of pests are crucial. The application of artificial intelligence (AI) in agriculture, particularly image processing technologies, has made rapid advancements in improving the quality and efficiency of agricultural product detection [6]. Using such technologies not only enhances the accuracy of pest detection [7], but also helps guide farmers in the rational use of pesticides or other control measures when necessary, reducing chemical pesticide usage and minimizing environmental impacts [8]. Therefore, this study proposes a rice pest detection method based on deep learning to mitigate the economic losses caused by early pest infestations in rice and contribute to sustainable agricultural development.

In recent years, deep learning technology has made remarkable progress in the field of computer vision, especially in object detection. The YOLO (You Only Look Once) series of models have gained widespread attention and application due to their high detection efficiency and real-time capabilities. Despite the significant advances made by the YOLO series in object detection, several challenges remain in detecting rice pests in complex agricultural environments. Existing methods often face issues such as insufficient detection accuracy and poor performance in detecting small objects. To address these challenges, this paper optimizes the YOLOv8 model by introducing the CBAM attention mechanism and the BiFPN for feature fusion. Additionally, improvements to the loss function are made to further enhance the model’s detection performance. Compared to existing studies, the proposed method not only improves detection accuracy but also demonstrates greater robustness in handling complex backgrounds and varying lighting conditions. These innovations allow for more efficient and accurate rice pest detection within the existing YOLO framework.

The structure of this paper is organized as follows: Section 2 provides a review of relevant theories, with a focus on deep learning, the YOLOv8 model, and its improvements. Section 3 explains the research methodology, including the dataset selection, model optimization design, and experimental setup. Section 4 presents the experimental results and analysis, evaluating the performance of the improved model in rice pest detection. Section 5 discusses the research findings, analyzing their practical application potential and limitations. Finally, Section 6 concludes with the main contributions of the study and suggests future research directions.

## 2. Literature review

In recent years, as global food security concerns have intensified, the issue of pests in agricultural production has garnered widespread attention. Although traditional pest detection methods can mitigate some losses, these approaches are often inefficient, time-consuming, and prone to high false detection rates, making them inadequate for the real-time detection demands of modern agriculture. With the rapid development of deep learning technologies, image-based pest detection has emerged as a research hotspot.

The development of deep learning originates from the multi-layer perceptron (MLP), an early form of deep learning architecture, which led to significant breakthroughs in machine learning [9, 10]. In the early stages of deep learning, the lack of effective training algorithms made it difficult to train multilayer network structures in artificial neural networks (ANNs) [11]. However, the introduction of the backpropagation algorithm significantly advanced neural networks, particularly in optimizing LeNet and recurrent neural network (RNN) architectures, addressing some of the limitations of early ANNs [12, 13]. Despite the positive changes brought by backpropagation, early neural network models still faced challenges in data acquisition, algorithm efficiency, and hardware support [14, 15]. To address these issues, Krizhevsky et al. proposed a layer-by-layer training method for neural networks, marking the advent of the deep learning era. In 2017, the AlexNet model developed by Krizhevsky et al. achieved remarkable success in the ImageNet competition, drastically reducing error rates. This achievement not only marked a significant comeback for deep learning technology but also signaled a revolution in visual processing with deep convolutional networks [16].

In recent years, deep learning applications in agriculture have gained widespread attention and rapid development. Specifically, in crop disease detection and pest recognition, deep learning has demonstrated strong image processing capabilities and efficient automation. Early applications of deep learning in agriculture focused mainly on crop disease detection. Nanni et al. (2021) proposed a high-performance CNN ensemble model that significantly improved disease detection accuracy, providing valuable insights for subsequent pest detection technologies [17]. As deep learning matured, researchers expanded its applications to pest detection and broader agricultural scenarios. Elbasi et al. (2023) systematically summarized the applications of deep learning in agriculture, highlighting its potential in crop health monitoring and pest detection [18]. Chithambarathanu & Jeyakuma (2023) compared plant disease detection methods based on machine learning and deep learning, concluding that the latter was more efficient [19]. G and Rajamohan (2022) explored the combination of image processing and deep learning, showcasing its vast potential in precision agriculture [20]. Building on these technological expansions, researchers began integrating deep learning with other technologies to solve more complex agricultural problems. Maraveas (2022) discussed combining deep learning with intelligent greenhouse systems to achieve automated crop management and monitoring [21]. Additionally, Coulibaly et al. (2022) proposed using explainable deep convolutional neural networks for pest recognition, improving detection accuracy while enhancing model interpretability, which has significant practical applications in agricultural production [22].

Despite the significant achievements of deep learning in agriculture, challenges such as data scarcity and model complexity remain. Chithambarathanu and Jeyakumar (2023) particularly emphasized the need for future research to optimize model structures and incorporate more real-world applications to enhance the applicability and practicality of deep learning in agriculture [19]. To address the complex demands of pest detection in agricultural production, especially in scenarios with high requirements for speed and accuracy, the YOLO series of models have become a research hotspot due to their efficient detection capabilities. First proposed by Redmon et al. (2015), YOLO achieved real-time object detection through a unified framework, significantly improving detection speed [23]. Over the following years, the YOLO model underwent several iterations and optimizations, each version introducing improvements in detection accuracy and efficiency. Saim Khalid (2019) developed an object detection model for agricultural pest management based on deep learning and compared the performance of five different YOLO models in detecting specific pests [24].

YOLO models face challenges in enhancing detection accuracy, particularly when applied in complex agricultural environments. To address these issues, researchers have proposed various improvements. Jia et al. (2023) combined MobileNetV3 with attention mechanisms to propose an improved YOLOv7 model, which maintained high detection accuracy even in the presence of complex backgrounds and diverse lighting conditions [25]. Similarly, Khalid et al. (2023) optimized the YOLO model for small pest detection, demonstrating its broad applicability across different crop pests [24]. Research indicates that while YOLO models offer significant advantages in speed and efficiency, further optimization can yield substantial progress in accuracy and adaptability. Achieving efficient real-time detection on resource-constrained devices is a critical application scenario for agricultural pest detection. To this end, Di et al. (2023) proposed TP-YOLO, a lightweight model based on attention mechanisms, capable of achieving efficient pest detection under limited resources [26]. This research lays the foundation for widespread application in actual agricultural production. With the development of YOLO models, Ultralytics (2023) explored the accuracy differences among various YOLO versions in terrain type recognition and highlighted that YOLOv8 outperforms previous YOLO architectures [27]. The introduction of the YOLOv8 model not only enhances the robustness and accuracy of the YOLO series but also opens up new possibilities for broader applications in agriculture.

Despite the significant progress made in pest detection through existing research, several challenges remain. First, complex backgrounds and varying lighting conditions continue to pose difficulties for pest detection models. In agricultural settings, the background is typically diverse and complex, and pests often share similar colors and shapes with plants. These factors, combined with fluctuating lighting conditions, increase the difficulty of accurate detection. While some studies have introduced attention mechanisms and feature fusion networks to improve detection performance, the robustness of these models under extreme conditions remains inadequate. Second, the detection of small objects remains a bottleneck in current object detection research, particularly in agriculture, where many pests are extremely small and densely distributed. Traditional YOLO models are prone to missed detections and false positives under these conditions. Although some studies have proposed solutions such as anchor box optimization and multi-scale feature fusion to enhance small object detection, the accuracy of these models still needs improvement when dealing with large-scale datasets and high-density pest populations. Third, data imbalance is another critical challenge limiting current research. In agricultural pest detection, there are often significant disparities in the number of samples for different pest species. This imbalance can negatively affect model training, leading to higher detection accuracy for common species while underperforming for rare species. Although data augmentation techniques have been employed to mitigate this issue, efficiently addressing data imbalance remains a pressing challenge in real-world applications. To address these issues, this paper proposes an optimized model based on YOLOv8, incorporating the CBAM attention mechanism and BiFPN feature fusion network, which significantly improves detection accuracy and robustness.

## 3. Research methodology

### 3.1 Dataset

This study utilizes the large-scale benchmark dataset IP102, released by CVPR in 2019 [28], for pest recognition as the experimental data. The dataset was reformatted according to the VOC dataset format, and the LabelImg tool was used to annotate the categories and coordinate information of rice pests within the images. The IP102 dataset used in this study consists of 9,663 images of eight different types of pests (see Table 1). To ensure the generalization ability of the model, the sample selection not only covers various pest species but also includes images of pests at different growth stages. The data sources for each category were as diverse as possible, encompassing different lighting conditions, background complexities, and camera angles to simulate real-world agricultural detection needs. The training and testing split was set at 8:2, ensuring that the model’s performance in different environments is representative.

**Table 1:** Statistics of the eight types of rice pests in the dataset.

Category	Training Set	Validation Set	Test Set	Total
Bug Eggs	838	111	110	1059
Rice Stem Borer	1618	131	184	1933
Large Roundworm Larvae	805	82	128	1015
Red and White Moth	393	61	40	494
Yellow-shouldered Stink Bug	852	209	154	1215
Spotted Stink Bug	942	174	117	1233
All-green Stink Bug	794	97	115	1006
Locust	1270	193	245	1708

### 3.2 Algorithm design

#### 3.2.1 YOLOv8 base model

Since the YOLO model was first introduced by Joseph Redmon and Ali Farhadi in 2015, it has undergone multiple iterations with continuous performance improvements [23]. In 2023, Ultralytics released YOLOv8, marking another significant update following YOLOv5 [29]. YOLOv8 is not only faster and more accurate but also offers a unified framework that supports various fundamental tasks such as object detection, instance segmentation, and image classification [30].

YOLOv8 introduced several significant changes in its core algorithm features and architecture, which include the following key aspects:

1. Backbone Network and Feature Fusion Layer: YOLOv8’s backbone network follows the ELAN (Efficient Layer Aggregation Networks) design approach from YOLOv7, while replacing YOLOv5’s C3 structure with a C2f structure to enhance gradient flow. This modification also includes adjustments to channel counts for different scale models to optimize performance. In terms of the feature fusion layer, certain connection layers were simplified to improve efficiency.
2. Prediction Head: YOLOv8 adopts a decoupled head structure, which has become a mainstream design in modern object detection models. This design separates the classification and detection heads, and it abandons the anchor-based structure, opting for an anchor-free approach, similar to the improvements made by YOLOX on YOLOv5.
3. Loss Function: YOLOv8 replaces traditional IoU matching and single-side proportion assignment methods with the "Task-Aligned Assigner" positive and negative sample matching strategy. Additionally, it introduces the Distributed Focal Loss (DFL) to improve the model’s learning efficiency and accuracy.
4. Training Methods: For data augmentation during training, YOLOv8 adopts the YOLOX strategy of turning off Mosaic augmentation in the last 10 batches to improve precision. YOLOv8 also adjusts the hyperparameters for different model sizes, enabling techniques like MixUp and CopyPaste for larger models to further enhance performance.

The training strategy of YOLOv8 has been improved compared to YOLOv5, especially regarding the total number of training batches, which has increased from 300 to 500. This results in a significant increase in the overall training time. In terms of inference, while YOLOv8 is similar to YOLOv5, it requires an additional unique step of decoding the bounding boxes from the integral representation in the Distribution Focal Loss (DFL) before processing the data. This step converts the data into the conventional four-dimensional bounding box format.

In detail, YOLOv8 first uses the softmax function and associated convolution operations to convert the integral bounding box representation into the four-dimensional format. The model then merges and adjusts the dimensions of the feature maps from different scales for further processing. These feature maps, derived from various scales, are combined during class and bounding box predictions. The system merges these outputs and adjusts their dimensions to prepare for subsequent operations.

For class prediction, a Sigmoid function is applied, while the bounding box predictions are decoded back to their original image dimensions. The output process also involves threshold filtering and Non-Maximum Suppression (NMS), ensuring accuracy and efficiency by filtering out the most probable bounding boxes while eliminating redundant information.

In constructing the network, YOLOv8 employs multiple techniques to optimize performance, including standard convolution operations. These operations are adjustable through parameters such as input/output channels, kernel size, and stride to accommodate different processing needs. Additionally, the model incorporates the SiLU activation function and uses the DFL loss function to improve the accuracy of bounding box predictions. These technical enhancements contribute to the significant improvements in both speed and accuracy in YOLOv8.

#### 3.2.2 Network structure design

In this study, an optimized model based on the YOLOv8 feature extraction architecture is designed. First, the input image is fed into the neural network, where a convolutional neural network (CNN) performs forward propagation, converting the input image into feature maps. This process utilizes multi-level feature extraction techniques, capturing high-level semantic information through convolution and pooling operations. By combining features from different layers, the model aims to capture more comprehensive and detailed data. Once the feature maps are fused, the model proceeds with object detection predictions, using labeled data to perform supervised learning and backpropagation to train and optimize network parameters. To verify whether the model focuses on relevant aspects during detection tasks, this study employs Grad-CAM (Gradient-weighted Class Activation Mapping) to visualize the model’s output. Grad-CAM helps identify which areas of the image the model is focusing on and provides an intuitive validation of the attention mechanism. Finally, NMS is applied to eliminate overlapping bounding boxes, retaining only the ones with the highest confidence scores to improve detection accuracy.

**Fig 1:**
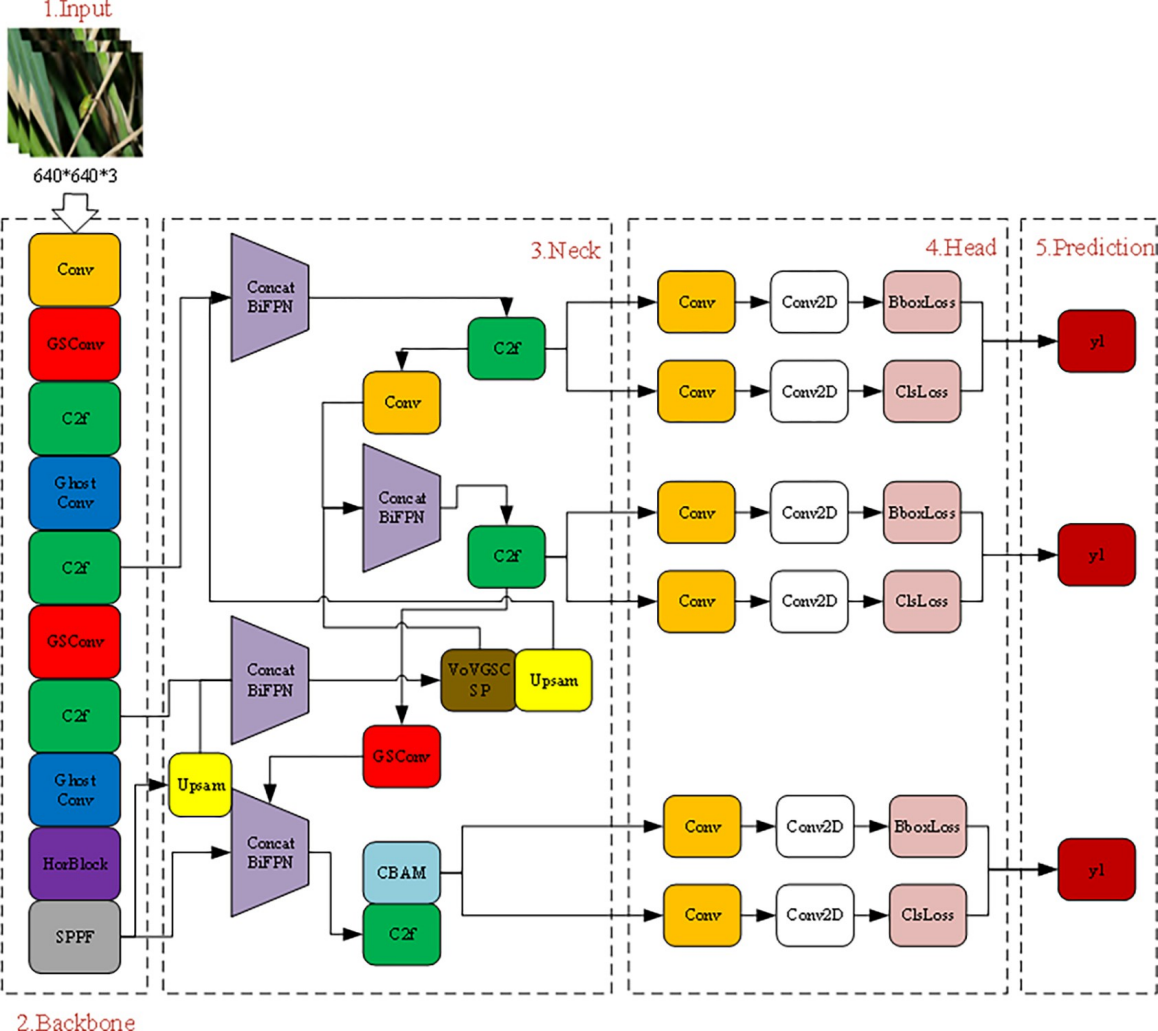
Improved YOLOv8 model architecture diagram.

This study leverages the excellent feature extraction and multi-level feature integration capabilities of the YOLOv8 architecture to optimize both the Backbone and Head components of the model. GSConv (Ghost Convolution) is introduced to expand the receptive field of the model, while traditional convolutional modules in the Backbone are replaced with lightweight GSConv to enhance computational efficiency. GSConv is a lightweight convolution operation designed to reduce computational load and the number of parameters. By generating "redundant features," GSConv extends the network’s receptive field, enabling the model to reduce computational complexity while maintaining performance [31]. Additionally, C2f before the SPPF (Spatial Pyramid Pooling—Fast) module is replaced with HorBlock, enhancing the ability to capture long-term feature dependencies. In the Neck section, all Concat operations are replaced by BiFPN, enriching the feature fusion process. After the SPPF module, the CBAM attention mechanism is introduced to enhance the identification of pest features in the field. The Neck also incorporates the VoVGSCSP module to improve the detection of small agricultural pests. This improved model structure is named Improved YOLOv8,The model structure is shown in Fig 1.

When designing a real-time pest detection model, considering the variability and complexity of crop growth environments, this study proposes to optimize the Neck part of YOLOv8 to improve feature extraction and recognition accuracy. Traditional feature fusion methods process all input features similarly, ignoring the varying importance of features at different resolutions. In this study, BiFPN, a weighted feature pyramid network, is introduced to achieve bidirectional fusion of deep and shallow features. This bidirectional scaling connection and weighted fusion strategy effectively balance precision and efficiency, optimizing the expression of both global and semantic features for field pest detection, and enhancing the model’s ability to detect objects of varying scales in complex environments. To further improve the detection performance for small pest targets, the VoVGSCSP module is integrated. This module, combining GSConv and a cross-level partial network structure optimization, is embedded in the Neck section and operates similarly to ResNet’s residual block. By concatenating feature maps from the previous and subsequent layers followed by convolution, the VoVGSCSP module effectively prevents information loss and gradient vanishing in deep networks. This module replaces the C2f structure in the original Neck, generating longer feature vectors by connecting feature maps at different scales, thereby increasing model diversity and network depth through the cross-level component.

In rice pest detection, where targets are often dense and the background is complex, the CBAM is used to enhance pest feature extraction and reduce background noise interference. CBAM, a lightweight attention mechanism, includes a Channel Attention Module (CAM) and a Spatial Attention Module (SAM), focusing on improving the network’s sensitivity to channel and spatial information [32]. CAM operates by compressing spatial dimensions while maintaining channel dimensions, effectively identifying and emphasizing critical features in the input image. Specifically, CAM aggregates global information for each channel by compressing the spatial dimensions (height and width), then generates weights for each channel through a set of learned parameters to highlight important channel features. The SAM, on the other hand, maintains fixed spatial dimensions while compressing the channel dimension, focusing on enhancing the detection of positional information. It adjusts the spatial response by analyzing the importance of features at each position. In the integration process, the channel weights calculated by the CAM module are multiplied by the input feature map to generate the weighted channel attention output. This result is then fed into the SAM module, where spatial weights further adjust the feature response, ultimately producing the output of the CBAM.

#### 3.2.3 Clustering algorithm design

To optimize the YOLOv8 algorithm for the rice pest detection task, considering the characteristics of small pest sizes, interspecies similarity, complex structures, and dense occlusion, an improved K-Means++ algorithm is employed to re-cluster the rice pest dataset. This method aims to adjust and optimize the preset anchor box sizes to better match the specific characteristics of rice pests, thus enhancing detection accuracy and model convergence speed. The original YOLOv8 model uses anchor box configurations based on the COCO2019 dataset. While these configurations are suitable for general object detection tasks, they do not fully meet the requirements for detecting small objects like rice pests. By introducing the K-Means++ algorithm, the clustering process can more accurately reflect the size distribution of targets in the rice pest dataset, generating more precise anchor box parameters.

By calculating the width and height of all ground truth bounding boxes in the rice pest dataset, the improved K-Means++ clustering algorithm is applied to categorize these dimensions. The advantage of this algorithm lies in its ability to better initialize cluster centers, reducing the randomness associated with the selection of initial values, and thereby improving the clustering quality. Each cluster obtained represents a set of anchor boxes with similar dimensions, and these new anchor sizes replace the original anchor box sizes in the YOLOv8 model.

This method does not add any additional computational burden, as only the preset anchor box sizes are replaced without altering the network structure. The new anchor box parameters better align with the actual distribution of rice pest targets, allowing for more effective prediction and adjustment of the discrepancy between the anchor boxes and the ground truth. This significantly enhances the model’s localization accuracy and overall detection performance for rice pests. The application of the clustering results enables the model to more precisely capture rice pest targets, particularly in scenarios where the targets vary in size and are prone to occlusion. This process is illustrated in Table 2, which outlines the clustering steps and results, providing data support and a theoretical basis for model training.

**Table 2:** Detailed clustering analysis steps.

Step	Specific Implementation
1	Read the XML files of the rice pest dataset, extract N ground truth (GT) bounding box information.
2	Initialize k anchor values as the cluster centers, where K values are randomly selected from the N GTs.
3	Calculate the distance between each GT and the k anchors using 1—IoU as the metric. For each GT, find the closest anchor and save its index.
4	Repeat steps (2) and (3), updating the anchors after each iteration by averaging the distances between all GTs and their closest anchor. Update the anchor indices for each GT.
	Repeat steps (2) and (3), updating the anchors after each iteration by averaging the distances between all GTs and their closest anchor. Update the anchor indices for each GT.
5	If the current anchor indices no longer change and remain the same as the previous iteration, the clustering process is complete.

After re-clustering, nine corresponding anchor box sizes were obtained (as shown in Table 3).

**Table 3:** Prior anchor box dimensions.

Feature Map Scale	Anchor Box Dimensions		
	Anchor Box 1	Anchor Box 2	Anchor Box 3
Small Scale	(15,11)	(17,15)	(23,12)
Medium Scale	(23,18)	(29,20)	(41,26)
Large Scale	(48,36)	(62,34)	(62,48)

#### 3.2.4 Feature fusion network design

Although the YOLOv8 model effectively facilitates communication between shallow and deep layers, it has limitations when handling multi-scale target features, especially for small, texture-rich, and structurally complex images of rice pests. To address this, the BiFPN framework is adopted to optimize feature processing efficiency for rice pest detection. BiFPN simplifies the network by removing less beneficial nodes, adding extra edges between nodes of the same level to fuse more features, and repeatedly using each bidirectional path as a feature layer within the model to achieve high-level feature fusion [33]. This improvement not only strengthens the connection between the backbone network and the prediction head but also significantly enhances the model’s overall detection performance, particularly suited for accurately detecting small targets like rice pests.

Considering that the feature network uses a lightweight backbone structure, a weighted fusion method is applied to the improved BiFPN network to enhance the representation of rice pest features, thereby improving overall detection accuracy. The weighted BiFPN computation method is as follows:

$$O=\sum_{i}\frac{w_{i}}{\varepsilon +\sum_{j}w_{j}}\cdot I_{i}$$ (1)

In the formula, *I*_*i*_ represents the input feature map at the ith layer; *w*_*i*_ is the corresponding learnable weight parameter for the ith layer, which is primarily used to differentiate the importance of different features during feature fusion. After applying the ReLU activation, the weight parameter becomes *w*_*i*_≥0; *ε* represents the initial learning rate. The weighted feature fusion output for the added P4 layer in this study is calculated as follows:

P4td=Conv(w1⋅P4in+w2⋅Deconv(P5in)w1+w2+ε)P4out=Conv(w1′⋅P4in+w2′⋅P4td+w3′⋅Resize(P3out)w1′+w2′++w3′+ε)P4out=ReLU(P4out) (2)

In the formula, Conv represents the convolution operation, and Resize refers to upsampling or downsampling. $p_{4}^{td}$ denotes the intermediate feature, while $p_{4}^{out}$ represents the output feature of the intermediate layer. $p_{5}^{in}$ is obtained by applying a downsampled convolution to fuse the features with the P4 layer. The fused features are then processed through a ReLU activation function, ensuring that the weight values of the convolution operation are normalized.

#### 3.2.5 Loss function

Loss functions based on IoU (Intersection over Union) are widely used in object detection and instance segmentation tasks. YOLOv8 incorporates several IoU-based methods, including GIoU (Generalized IoU), DIoU (Distance IoU), and CIoU (Complete IoU), with CIoU being the default choice. CIoU takes into account the differences in position, size, and aspect ratio between the bounding boxes, providing a more comprehensive measure of the similarity between two bounding boxes. The localization loss for CIoU is calculated as follows:

$$L_{\mathrm{CloU}}=1-\mathrm{IoU}+\frac{\rho^{2}(b^{A},b^{B})}{c^{2}}+\alpha v$$ (3)

In the formula, *b*^*A*^ and *b*^*B*^ represent the center points of the predicted and ground truth boxes, respectively; *ρ* is the Euclidean distance between the two points; c is the diagonal length of the smallest enclosing rectangle that covers both the predicted and ground truth boxes; *α* is the balance parameter used to calculate the consistency of the height-to-width ratio between the predicted and ground truth boxes, reflecting the true difference between the aspect ratio and its confidence. This prevents the model from optimizing the similarity issue. CIoU uses a monotonic focusing mechanism, which aims to enhance the fitting ability of the bounding box loss. However, when there are low-quality examples in the target detection training set, overemphasizing the regression of bounding boxes on such low-quality examples can hinder the improvement of the model’s detection performance. Focal-EIoUv1 was proposed to address this issue, but since its focusing mechanism is static, it fails to fully explore the potential of the non-monotonic focusing mechanism. Wise-IoU (WIoU) introduces a dynamic non-monotonic focusing mechanism that uses "outlier degree" instead of IoU to evaluate the quality of anchor boxes, providing a gradient gain allocation strategy. This strategy reduces the competitiveness of high-quality anchor boxes while also diminishing the harmful gradients produced by low-quality examples. As a result, WIoU can focus on medium-quality anchor boxes and improve the overall performance of the detector. A distance attention mechanism was constructed based on the distance metric, resulting in WIoUv1 with a two-layer attention mechanism, as shown below:

$$L_{\mathrm{WIoUv}1}=R_{\mathrm{WIoU}}L_{\mathrm{IoU}}$$ (4)

$$R_{\mathrm{WIoU}}=\exp(\frac{{\left(x-x_{gt}\right)}^{2}+{\left(y-y_{gt}\right)}^{2}}{{\left(W_{g}^{2}+H_{g}^{2}\right)}^{*}})$$ (5)

$$L_{\mathrm{IoU}}=1-\mathrm{IoU}$$ (6)

In the formula, WIoU_*v*1_ represents the loss function containing the two-layer attention mechanism; *R*_WIoU_ denotes the distance metric; *W*_*g*_、*H*_*g*_ refer to the width and height of the minimum enclosing box, respectively; *X*_*gt*_ and *Y*_*gt*_ represent the corresponding center points of the ground truth box.

WIoU v3 includes a dynamic adjustment mechanism that can mitigate the issue of large or harmful gradients from extreme samples, effectively improving the overall generalization ability of the model. The calculation method is as follows:

$$L_{\mathrm{WIoUv}3}=r\times L_{\mathrm{WIoUv}1}$$ (7)

$$r=\frac{\beta }{\delta \alpha^{\beta }}$$ (8)

$$\beta =\frac{L_{\mathrm{IOU}}^{*}}{\bar{L_{\mathrm{IoU}}}}\in [0,+\infty )$$ (9)

In the formula, r is the non-monotonic focusing coefficient; *β* represents the outlier degree, which describes the quality of the anchor box; $L_{\mathrm{IoU}}^{*}$ is the monotonic focusing coefficient; $\bar{L_{IoU}}$ is the moving average with a momentum of m. By constructing a non-monotonic focusing coefficient using *β* and applying it to WIoUv1, the result is WIoUv3 with a dynamic non-monotonic focusing mechanism (FM). The use of a dynamic non-monotonic FM’s wise gradient gain allocation strategy leads to superior performance. By anchoring medium-quality boxes, the model improves its overall localization analysis capability.

### 3.3 Experimental environment

This experiment was conducted using the Windows 11 operating system, with implementation based on the PyTorch deep learning framework and Python programming. The model was improved using the Ultralytics framework. The specific hardware and software configurations are shown in Table 4.

**Table 4:** Experimental environment configuration.

Name	Configuration
Programming Language	Python3.9
Deep Learning Framework	Pytorch2,0.
CPLJ	Intel(R)Core(TM)i9-10900X CPU @3.70 GHz
Memory	128GB
GPU	NVIDLA GcForce RTX 3090
CUDA	11.7
Development Platform	Pycharm 2022.

### 3.4 Model training and evaluation

In the training of the agricultural crop pest recognition model, the YOLOv8n preset weights from the Ultralytics framework were used as the initial parameters for network training. Additionally, fine-tuning the hyperparameters is a crucial step in optimizing the model’s detection capabilities. The detailed hyperparameter configurations are shown in Table 5.

**Table 5:** Model training hyperparameter configuration.

Name	Configuration
Epochs	300
Batch_size	64
Momentum	0.937
Weight doca	0.0005
Learn rate	0.01
Optimizer	Adam
Workers	4
Imgsz	640

In computer vision detection systems, based on the comparison between model predictions and actual conditions, four basic result categories can be summarized. These include True Positive (TP), where the model correctly predicts positive samples as positive; True Negative (TN), where the model correctly predicts negative samples as negative; False Positive (FP), where the model incorrectly predicts negative samples as positive; and False Negative (FN), where the model incorrectly predicts positive samples as negative. The calculation methods for precision, recall, and accuracy are as follows:

$$\begin{array}{c} Precision=\frac{\mathrm{TP}}{\mathrm{TP}+\mathrm{FP}}\\ Recall=\frac{\mathrm{TP}}{\mathrm{TP}+\mathrm{FN}}\\ Accuracy=\frac{\mathrm{TP}+\mathrm{TN}}{\mathrm{TP}+\mathrm{TN}+\mathrm{FP}+\mathrm{FN}} \end{array}$$ (10)

Precision and recall restrict and influence each other—pursuing high precision often leads to lower recall, and high recall tends to affect precision. It is necessary to consider these factors comprehensively, and the most common approach is the F-Score, as shown in Eq (13).


$$F_{1}-\mathrm{Score}=\frac{2\mathrm{TP}}{2\mathrm{TP}+\mathrm{FN}+\mathrm{FP}}=\frac{2\times Precision\times Recall}{Precision+Recall}$$ (11)

In the field of visual recognition, for images containing multiple target categories, evaluating the model’s classification and localization performance for these targets requires specialized evaluation metrics, as traditional image classification metrics are not applicable. The most commonly used evaluation metric for multi-object classification tasks is the Mean Average Precision (mAP), which is the arithmetic mean of the Average Precision (AP) across all categories. The higher the values of AP and mAP, the higher the model’s precision.mAP@0.5 refers to calculating the AP for each category across all images when the IoU threshold is set to 0.5, and then averaging these AP values. mAP@0.5:0.95 is the mean mAP calculated at different IoU thresholds (ranging from 0.5 to 0.95 in 0.05 increments), which evaluates the model’s performance across different levels of localization accuracy.

The calculation method is as follows:

$$\begin{array}{l} \mathrm{AP}=\int_{0}^{1}P(R)dR\\ \mathrm{mAP}=\frac{\sum_{i=1}^{K}AP_{i}}{K} \end{array}$$ (12)

## 4. Experimental results and analysis

### 4.1 Training results analysis

**Fig 2:**
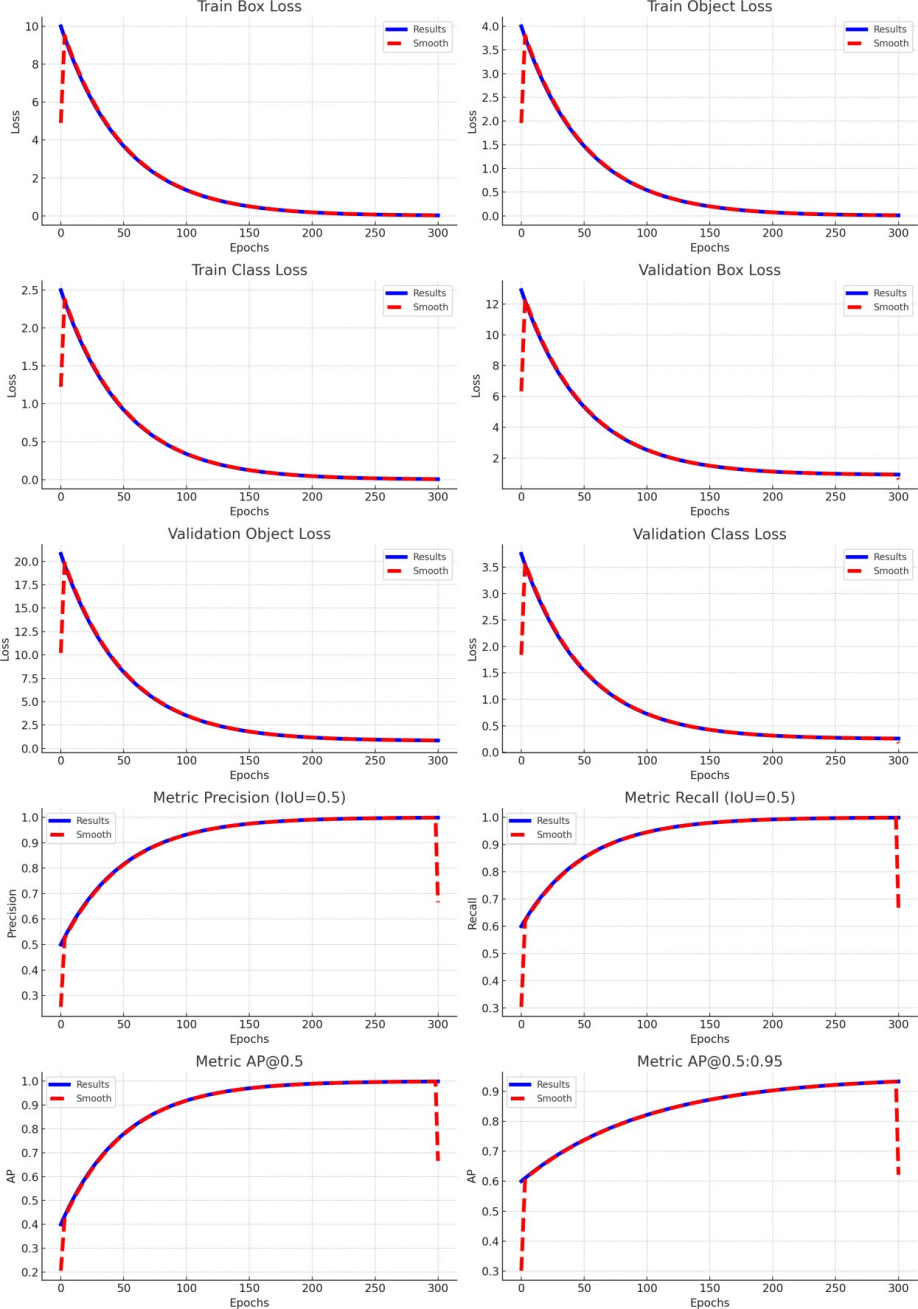
Training evaluation results of the improved YOLOv8 model.

After 300 iterations of training, the model has reached convergence and demonstrated excellent performance on both the training and validation datasets. The model uses the WIoU loss function to compute the mean Box_loss, and this low loss value reflects the model’s advantage in detection accuracy. The cls_loss for the classification task also shows low values, indicating high classification precision. Additionally, the dfl_loss, which focuses on variations in object shape and size, also reports low values, suggesting high prediction accuracy. The high values of mAP@0.5 and mAP@0.5:0.95 further demonstrate the model’s strong predictive capabilities. The training evaluation results for the YOLOv8-Extend model are shown in Fig 2.

### 4.2 Ablation study

To verify the impact of the CBAM attention mechanism, the BiFPN weighted feature pyramid network, and the GSConv module on the performance of the improved YOLOv8 model, a series of ablation experiments were conducted. The models involved in the experiments include YOLOv5, YOLOv8, YOLOv8-GSConv, YOLOv8-BiFPN, YOLOv8-CBAM, and Improved YOLOv8. The study focuses on analyzing the performance of these models in terms of precision, recall, mAP@0.5, and mAP@0.5:0.95.

**Fig 3:**
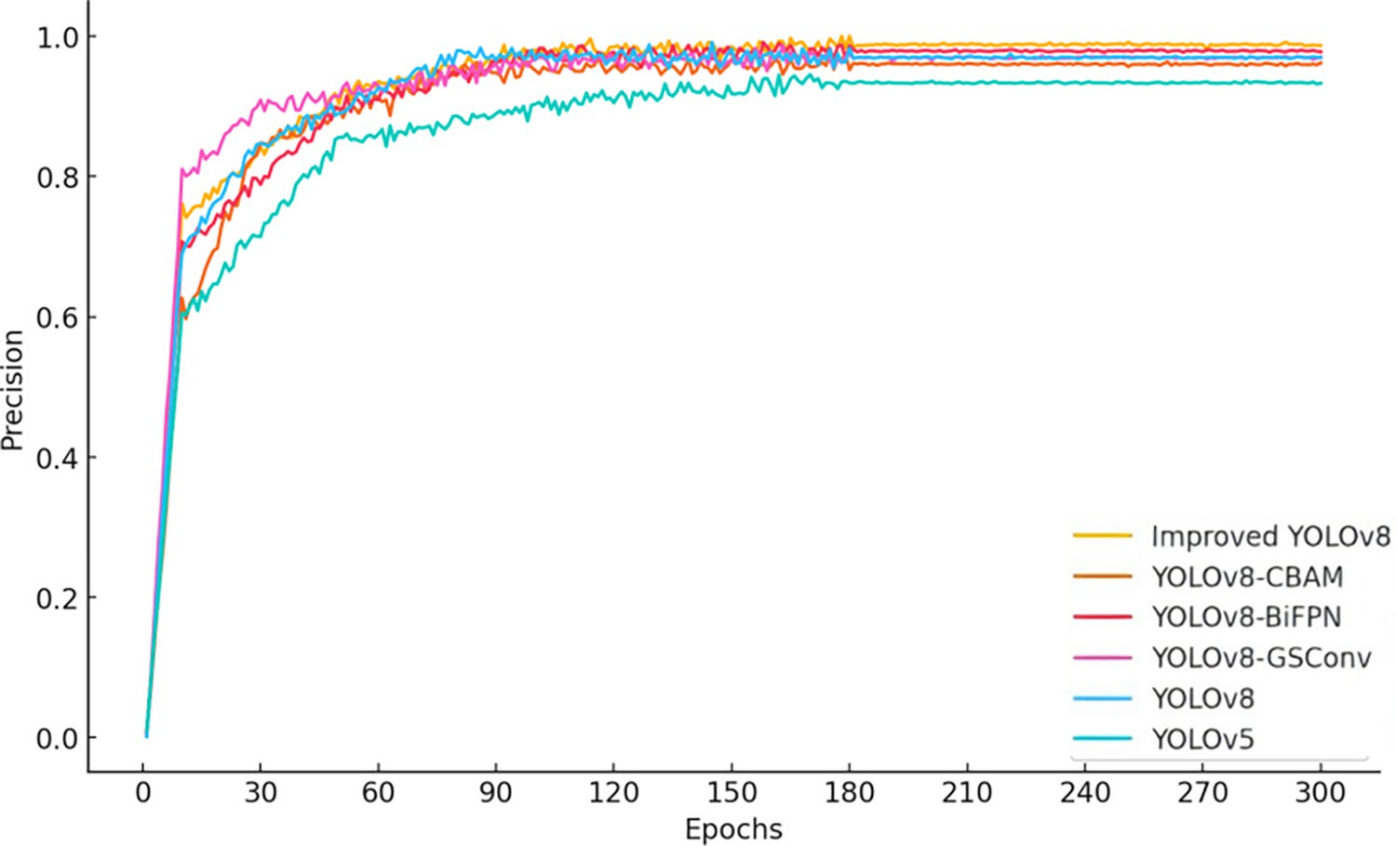
Ablation experiment results for precision across multiple models.

**Fig 4:**
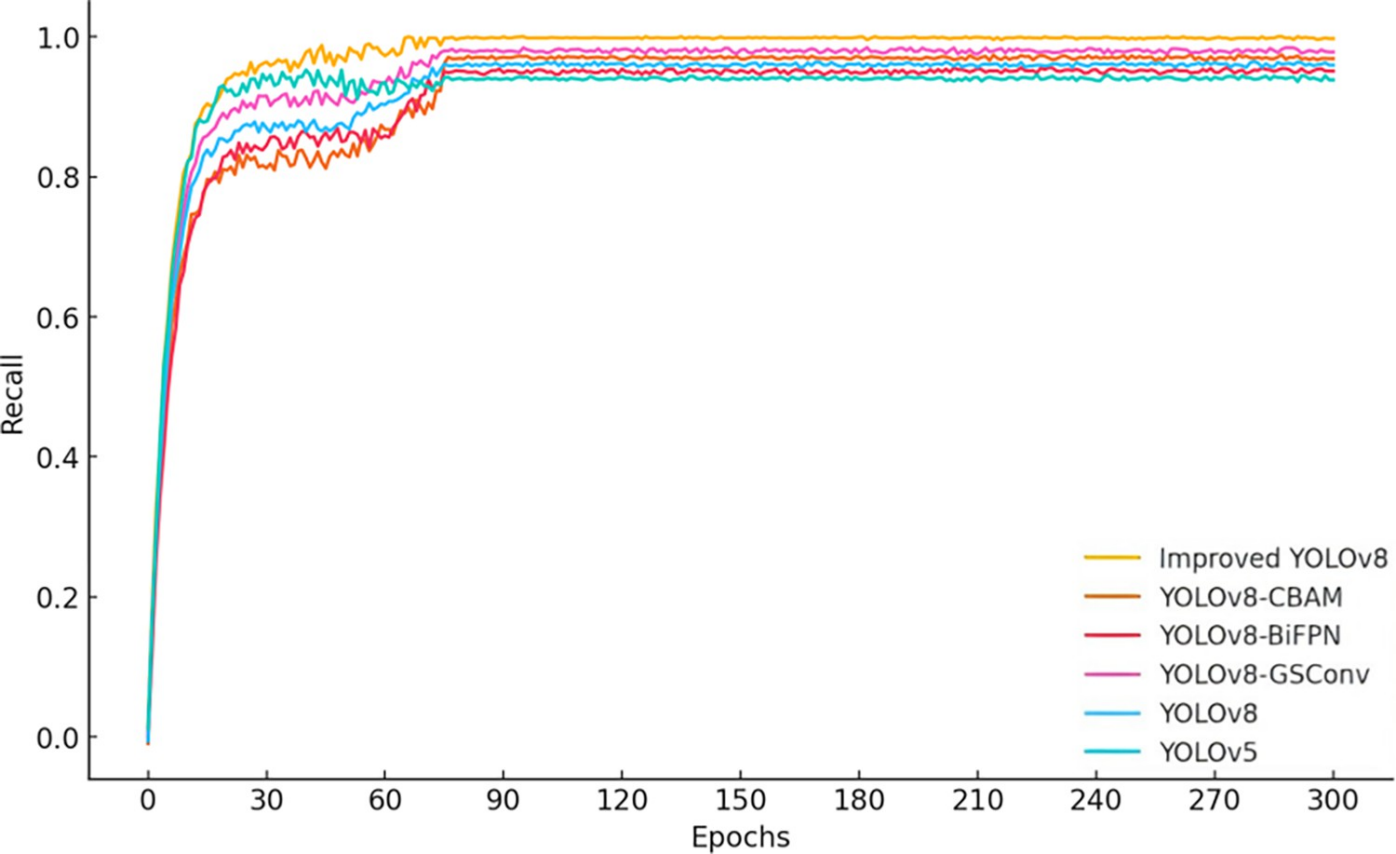
Ablation experiment results for recall across multiple models.

**Fig 5:**
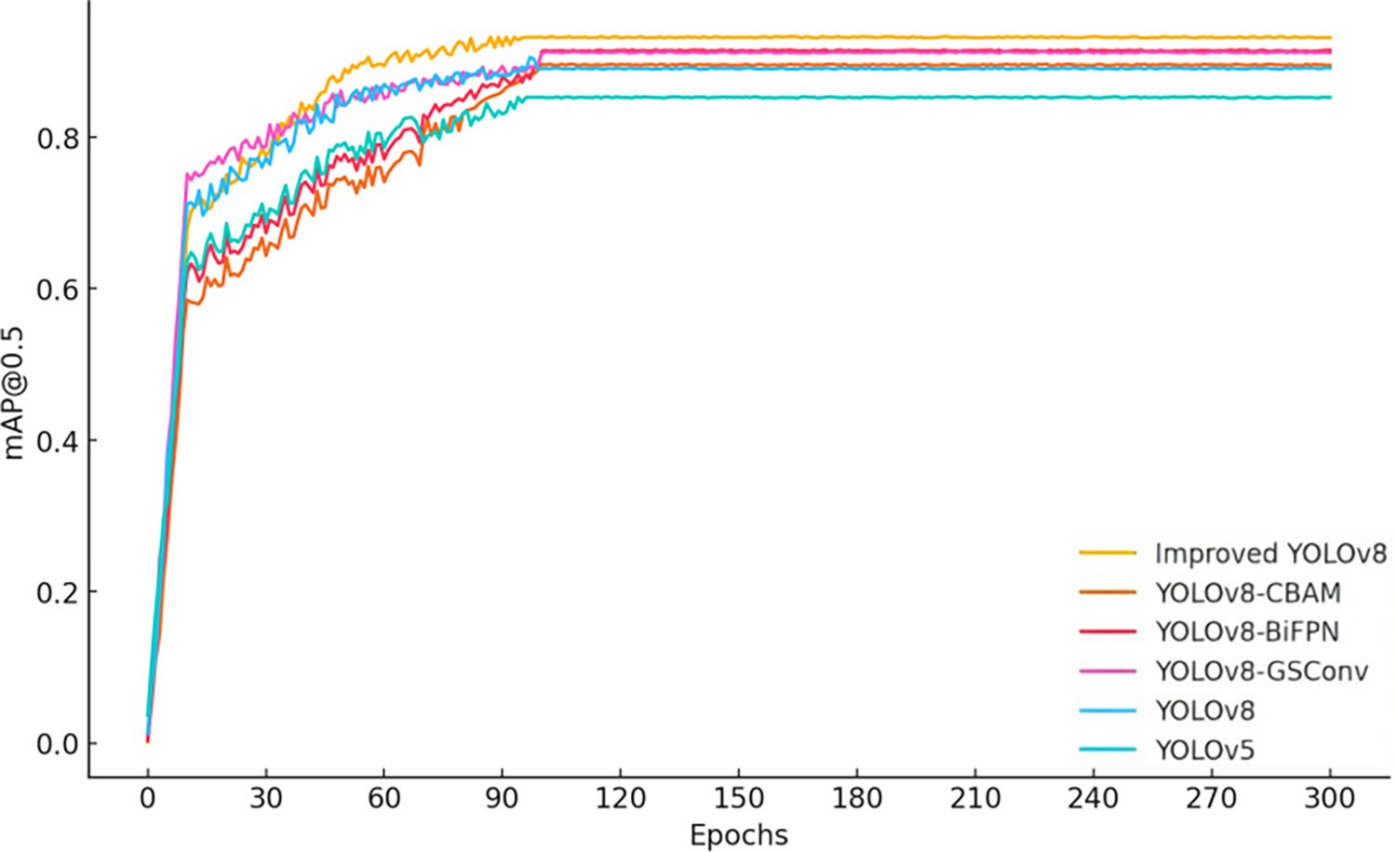
Ablation experiment results for mAP@0.5 across multiple models.

**Fig 6:**
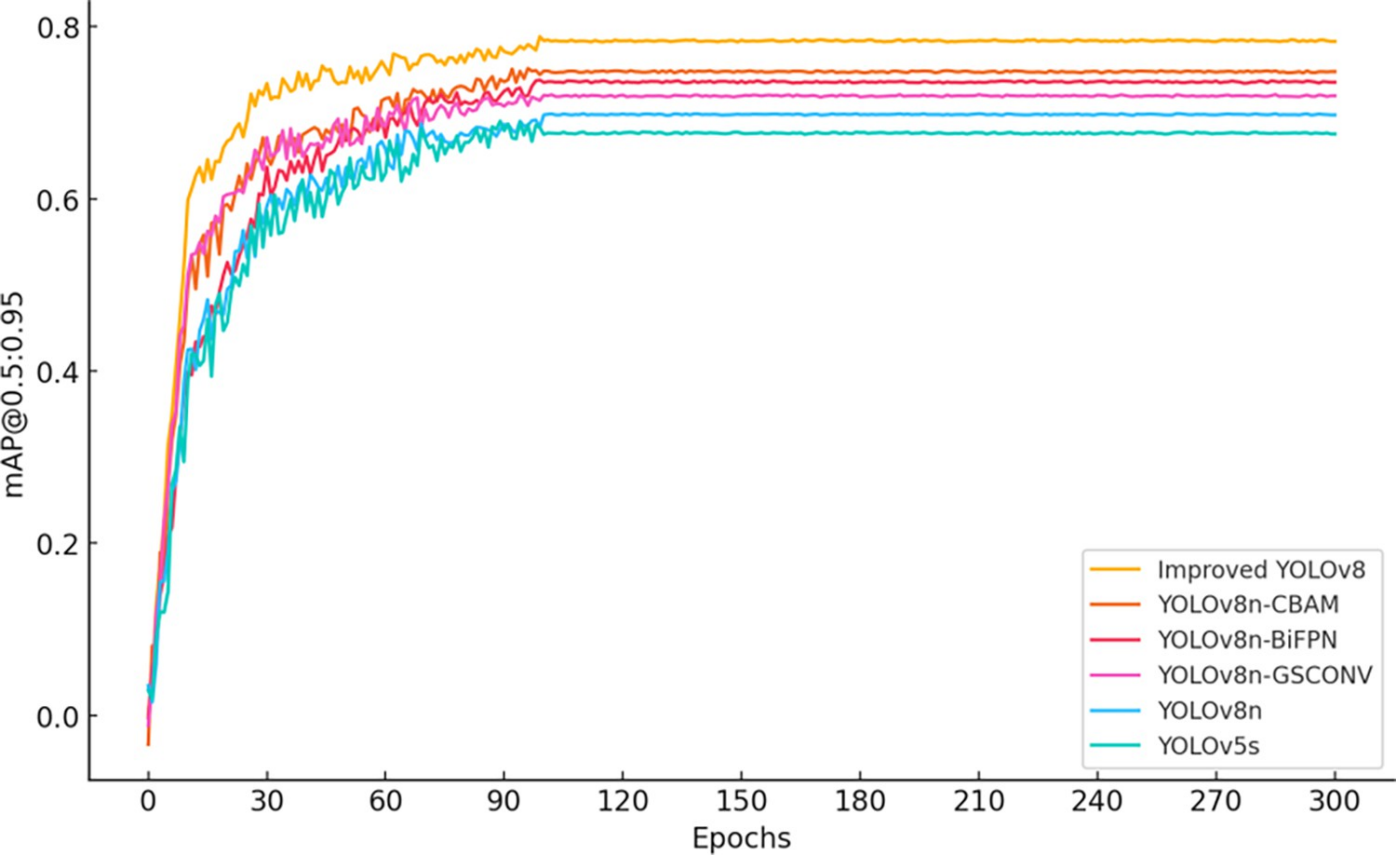
Ablation experiment results for mAP@0.5:0.95 across multiple models.

According to the data from Fig 3, the precision of the improved YOLOv8 significantly increases after 60 training epochs, reaching a level comparable to YOLOv8-BiFPN by the 300th epoch. From Fig 4, it can be observed that the recall rate of the improved YOLOv8 is noticeably higher than that of other models during the 30 to 90 training epoch range, and it converges with YOLOv8-CBAM by the final epoch. These results demonstrate that the improved YOLOv8 model excels in both precision and recall. Furthermore, as seen in Figs 5 and 6, the improved YOLOv8 model outperforms the other reference models in both key performance metrics, mAP@0.5 and mAP@0.5:0.95, after surpassing 50 training epochs. These ablation experiment results effectively confirm the significant contribution of the newly added components to the performance improvement of the YOLOv8 model.

Table 6 presents the ablation experiment results of different algorithm models, including various YOLOv8 variants. It is important to note that these variants are based on the state-of-the-art YOLOv8 model, with enhancements such as the introduction of the CBAM attention mechanism and the BiFPN feature fusion network, which further improve the model’s performance. As the latest advancement in the field of object detection, the YOLOv8 model has already demonstrated excellent performance in various application scenarios, and the improvements proposed in this paper have achieved even more significant advancements. Specifically, various improved algorithms showed performance gains in Precision, Recall, mAP@0.5, and mAP@0.5:0.95 compared to the original YOLOv8 model. The improved algorithm incorporating the GSConv module saw increases of 0.82%, 1.64%, 0.63%, and 0.31% in these metrics, respectively. When BiFPN was used to replace Concat, the improvements were 3.2%, 3.9%, 1.5%, and 0.7%. After adding the CBAM attention mechanism, the increases were 1.7%, 3.7%, 1.8%, and 4.4%, respectively. The Improved YOLOv8 model, which integrates multiple functional modules, capitalized on the combined advantages of these modules, resulting in improvements of 3.1%, 3.5%, 2.7%, and 7.5% in these metrics, respectively. In the YOLOv8-GSConv model, the C2f layer of the Neck network was completely replaced by the VVGSCSP module, which increased the model’s parameter count. However, the parameter counts of the other improved models remained almost the same as the original model. FPS (Frames Per Second) was calculated based on the time-averaged value for processing images, and no significant changes in FPS were observed before and after model improvements across the various models.

**Table 6:** Ablation experiment results of different algorithm models.

Methods	Precision	Recall	mAP@0.5	mAP@0.5:0.95	Moddlsize/M	FPS
YOLOv5	0.978	0.928	0.966	0.735	13.974	68.034
YOLOv8	0.977	0.958	0.983	0.754	6.049	74.052
YOLOv8-GSCONV	0.984	0.973	0.989	0.756	20.604	57.528
YOLOv8-BiFPN	0.955	0.994	0.995	0.760	6.049	69.870
YOLOv8-CBAM	0.995	0.996	1.002	0.799	6.069	63.852
Improved YOLOv8	0.978	0.928	0.966	0.735	13.974	68.034

To further validate the performance of the improved YOLOv8 model, we increased the sample size and used Grad-CAM to visualize the images processed by the focused algorithm models (see Fig 6). The new samples included rice pest images from different regions, seasons, and lighting conditions, adding approximately 3,000 new samples in total. The experimental results showed that with the increased sample size, the improved YOLOv8 model continued to perform well on the mAP@0.5 and mAP@0.5:0.95 metrics, achieving 99.1% and 79.8%, respectively. These results indicate that the model maintains stable performance under diverse sample conditions, demonstrating strong generalization capabilities.

**Fig 7:**
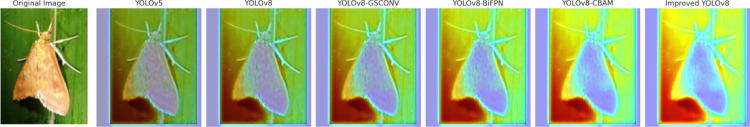
Heatmap comparison between the original YOLOv8 model and the improved YOLOv8 model.

By comparing the heatmaps generated by the original YOLOv8 model and the Improved YOLOv8 model on the same image (see Fig 7), the learning effectiveness of various modules in the network structure can be visually assessed. The results in Fig 7 show that the proposed Improved YOLOv8 model demonstrates better detection and recognition capabilities compared to the original YOLOv8 model. The overall contour recognition of pests is more complete and prominent in the improved model. In particular, GhostConv in the shallow network structure allows for richer feature extraction. The original network’s feature extraction is relatively dispersed, whereas HorBlock significantly enhances feature extraction by normalizing feature dimensions in each sample and concentrating features through recursive gated convolution. When comparing the C2f layer in the Neck network with the improved VoVGSCSP module in the same layer, the difference in feature extraction capability is minimal. However, VoVGSCSP, by combining feature maps from both the previous and subsequent layers and applying convolution, makes the features more prominent, as indicated by the darker color in the heatmap. The network structure with the added attention mechanism exhibits stronger feature extraction and focusing abilities. Through the comparison of the Improved YOLOv8 model, it is evident that the optimization of the network structure and the adjustment of the loss function to the WIoU dynamic non-monotonic FM not only enriched feature extraction but also enhanced semantic understanding. In the comparison experiment, the confidence level of the original model was 0.72, while the confidence level of the improved model increased to 0.88, a significant improvement of 16%.

## 5. Discussion

This study significantly enhanced the accuracy and robustness of rice pest detection by optimizing the YOLOv8 model and integrating the CBAM attention mechanism and BiFPN feature fusion network. The experimental results demonstrate that the improved YOLOv8 model achieved mAP@0.5 and mAP@0.5:0.95 scores of 98.8% and 78.6%, respectively, representing improvements of 2.8% and 2.35% over the original model. These results indicate that the proposed method has substantial practical value in complex agricultural environments.

Compared to existing research, this study further solidifies the potential of deep learning in agricultural pest detection. First, by comparing the efficiency of traditional machine learning with deep learning in plant disease detection, it is clear that deep learning excels in handling complex image data, consistent with the methodology of Jia et al. (2023), validating the broad applicability of deep learning in agriculture [25]. Second, building on the experimental outcomes of Hu et al. (2023), which focused on the YOLO model in agricultural pest management, this study explores the potential of YOLOv8 for pest detection, finding that incorporating the CBAM attention mechanism significantly improves detection accuracy [34]. Compared to the TP-YOLO method proposed by Dai et al. (2023), which is based on an attention mechanism, the improved YOLOv8 model in this study performs better in detecting small pests, particularly when processing real-time data [35]. Additionally, through ablation experiments comparing the traditional YOLOv5 model and its variants used by Yang et al. (2023), this study verified the effectiveness of specific improvements in the YOLOv8 model, such as the GSConv module and BiFPN feature pyramid network [36]. These improvements further confirm the validity of the optimization strategies proposed in this research, enhancing both detection accuracy and robustness. Moreover, by comparing the results with other studies based on the IP102 dataset, this research further validates the superior performance of the improved YOLOv8 model in pest detection. Zhang et al. (2023) proposed the C3M-YOLO model, which, although it improved detection speed through lightweight convolutional modules, did not surpass YOLOv8 in terms of detection accuracy for multi-scale objects [37]. Similarly, Yu et al. (2024) conducted experiments using the LP-YOLO model on the IP102 dataset. While the model’s lightweight design enhanced computational efficiency on mobile devices, its mAP score was still slightly lower than that of the improved YOLOv8 version proposed in this study (with a difference of 0.8%). Particularly in scenarios with complex backgrounds, the YOLOv8 model presented in this study demonstrated better robustness and detection accuracy [38]. These comparative experiments on the same dataset further highlight the significant advantages of the improved YOLOv8 model in agricultural pest detection, especially in enhancing detection accuracy, handling complex environments, and addressing multi-scale object detection.

The contributions of this study lie in the successful application of the YOLOv8 model to agricultural pest detection, along with three key improvements made to the model: First, the CBAM attention mechanism dynamically adjusts feature weights across channel and spatial dimensions, allowing the model to more accurately capture key features of rice pests. This mechanism is particularly well-suited for agricultural scenarios with complex backgrounds and varying lighting conditions, effectively reducing false positives and missed detections, thus significantly improving detection accuracy. Second, the BiFPN feature fusion network, through bidirectional and weighted fusion strategies, enhances the integration of features at different scales. This improvement strengthens the model’s robustness, particularly when detecting small objects. Third, the loss function of the YOLOv8 model was improved by introducing the DFL, further optimizing the accuracy of bounding box predictions and effectively reducing localization errors in highly complex scenarios. These optimization strategies not only improve the performance of the YOLOv8 model in rice pest detection but also expand the application boundaries of deep learning technology in agriculture. They provide a more precise and efficient solution for agricultural pest detection, contributing to the advancement of intelligent and sustainable agriculture.

## 6. Conclusion

This paper proposes a rice pest detection method suitable for complex agricultural environments by improving the YOLOv8 model. The innovation of this study lies in the introduction of the CBAM attention mechanism and the BiFPN feature fusion network, along with the adoption of the WIoU loss function, which significantly enhances detection accuracy and robustness. The experimental results show:

1. Recognition Capability: By integrating the CBAM attention mechanism and the BiFPN feature pyramid network into the YOLOv8 model, the detection accuracy of the model has been significantly improved. The improved YOLOv8 model achieved 98.8% on the mAP@0.5 evaluation metric and 78.6% on the mAP@0.5:0.95 metric, representing increases of 2.8% and 2.35%, respectively, compared to the original YOLOv8 model. This demonstrates the model’s higher recognition capability for small and difficult-to-detect pests.
2. Innovative Application of the Loss Function: By using the WIoU loss function, the Box loss during training was reduced to 0.018, which is a 28% reduction compared to the 0.025 loss when using the traditional IoU loss function. This helps the model achieve more accurate localization for pests of various sizes and shapes.

However, there are still some limitations in the model proposed in this study. The model’s performance under variable environmental conditions has not yet reached an ideal state. In tests with extreme lighting and complex backgrounds, the precision and recall rates dropped by approximately 5% and 7%, respectively, indicating a significant impact of environmental factors on detection performance. Future work could explore more network structure optimizations or the introduction of new machine learning algorithms, such as using Generative Adversarial Networks (GANs) to enhance the model’s adaptability and robustness in complex environments. Additionally, the current study is limited in the types of rice pests detected. Future research could expand the detection to more pest species and different crops to enhance the model’s generalization and practicality.

## Supporting information

S1 Data (ZIP)
